# Patent Ductus Arteriosus in Extremely Preterm Infants: Update on Current Diagnostic and Treatment Options

**DOI:** 10.1007/s11936-025-01101-6

**Published:** 2025-07-22

**Authors:** Kimberly Fernandez Trahan, Elaine L. Shelton, Maria Gillam-Krakauer

**Affiliations:** Division of Neonatology, Department of Pediatrics, Vanderbilt University Medical Center, 2200 Children’s Way, DOT, 11111, Nashville, TN 37232 USA

**Keywords:** Patent ductus arteriosus, Acetaminophen, Transcatheter ductus closure, Bronchopulmonary dysplasia, Extreme prematurity

## Abstract

**Purpose of Review:**

Patent ductus arteriosus (PDA) treatment strategies in the neonatal period differ widely. Variations in what constitutes hemodynamic significance (hsPDA) and scarcity of high-quality data on long-term outcomes has contributed to lack of standardization. Filling these knowledge gaps would impact clinical decision making.

**Recent Findings:**

Recent trials have not shown improvement in outcomes with early compared to expectant management. Targeted neonatal echocardiography (TnECHO) has facilitated timely intervention with encouraging outcomes. Acetaminophen use is increasing even in infants < 24 weeks. Advancements in percutaneous transcatheter occlusion, characterization of the unique expression of genes and ion channels of the ductus arteriosus, and attention to nonpharmacologic strategies are essential advances in PDA management.

**Summary:**

With increased utilization of TnECHO, clarification of the scope of transcatheter-based closures, further understanding of the genetic and molecular factors involved in ductal tone, and the appreciation of the off-target effects that medications and fluid balance can have on the DA, providing targeted, individualized PDA treatment is achievable. However, the development of innovative therapies to promote ductal closure is a necessity.

## Opinion Statement

In the extremely preterm infant population, management of a PDA requires a comprehensive approach with thoughtful decision-making to ensure the patient population most at need of ductal closure receives treatment, while also limiting the exposure to potentially harmful medications and invasive procedures. Though indomethacin prophylaxis has short-term benefits including reduction in rates of intraventricular hemorrhage (IVH) and pulmonary hemorrhage, the lack of improvement in long-term outcomes and the high rate of unnecessary medication exposure makes this approach less optimal. Instead, a targeted approach to PDA treatment should be undertaken.

To differentiate which infants would benefit from PDA treatment, repeated clinical assessments and serial echocardiograms that evaluate the degree of hemodynamic impact are critical. Specific monitoring of left atrial volume, direction of flow in the descending aorta, and markers of overall volume status such as ventricular output and end-diastolic volume is important as is evaluation of the evolution of shunt physiology given the dynamic nature of a PDA. With echocardiographic evidence of hemodynamic significance, escalation in the degree of ductal shunting, or worsening clinical status, pharmacologic treatment should be considered in the absence of contraindications. While the current pharmacologic options have similar efficacy, the more favorable side effect profile of acetaminophen may make this medication a more ideal choice across gestational ages. With a persistent PDA, repeated courses may be necessary, though the response rate to pharmacotherapy diminishes with subsequent courses, and other interventions such as transcatheter device closure can be considered.

Additionally, nonpharmacologic management should be a focus of this high-risk population, and implementation of certain strategies, such as higher PEEP, permissive hypercapnia, and limitations of large fluid and sodium loads in the early postnatal period may reduce the need for other interventions. Understanding the prenatal and postnatal exposures of each infant is also relevant when deciding whether to initiate treatment as certain medications and maternal conditions are associated with DAs refractory to treatment, and efforts should be made to limit the exposure to these medications when feasible.

## Introduction

Despite a half century of basic and clinical investigation, many questions remain regarding the optimal evaluation and treatment of patent ductus arteriosus (PDA) in premature infants. PDA studies have been plagued by inclusion of patients with high incidence of spontaneous closure, use of varying diagnostic and treatment parameters, and high rates of open-label treatment, making it difficult to provide clear conclusions about the potential benefits, timing, method of intervention, and target population for intervention. Neonatal hemodynamics offers promising tools to fill in the information gaps necessary to move the field forward. Ongoing conundrums in neonatology include reliable predictors of spontaneous closure, optimal selection of pharmacologic management, and timing of pharmacologic and procedural closure. As PDA has been associated with worse outcomes, particularly bronchopulmonary dysplasia (BPD) and mortality [1], it is prudent to continue investigating how to improve the approach to this common condition. This review will discuss the latest evidence from the last year, with a particular focus on advancements in diagnosis using neonatal targeted echocardiography (TnECHO), management of infants born < 24 weeks, and nonsurgical closure of PDA using percutaneous transcatheter occlusion.

### Diagnosis

PDA severity is a spectrum, and the point at which the shunt becomes symptomatic or detrimental to neonatal outcomes and could benefit from intervention remains unclear. Many PDAs of varying sizes will undergo spontaneous closure while others become hemodynamically significant (hsPDA) and lead to clinical instability [2]. While important to delineate the structural (e.g. size, shape) characteristics of the PDA itself, it is imperative to specify the associated hemodynamic effects when defining a hsPDA. This can be a challenging and subjective task given the lack of clinical consensus regarding the defining criteria of a hsPDA, the difficult nature of which has been highlighted in recent studies. Chesi et al. [1] showed that infants with a PDA that did not meet criteria for hemodynamic significance had a nearly 15-fold increased risk of mortality compared to those without a PDA and up to an 11-fold increased risk of mortality compared to those whose PDA had a clear hemodynamic impact, defined as ductal diameter ≥ 1.6 mm and echocardiographic evidence of either pulmonary overcirculation or systemic hypoperfusion. This finding could reflect longer exposure to a ductal shunt as only 18% of infants categorized as having a non-hsPDA received early treatment versus 62% of those with a hsPDA. It highlights the need for a better definition of hemodynamic significance and consideration for postnatal age, since it suggests that certain infants may have benefitted from early minimization of their ductal shunt.

With growing appreciation for neonatal hemodynamics, the usefulness and limitations of echocardiography will be elucidated. Classic PDA diagnostic measures such as ductus size and left atrial to aortic root ratio (LA/Ao) are being refined with a goal to measure volume shunt, a more accurate predictor of hemodynamic significance. The LA/Ao is a single plane measurement and assumes the left atrium is a perfect sphere, potentially underestimating volume, and is subject to poor inter-rater reliability. Karimi et al. [3] evaluated left atrial volume (LAV) indexed to body surface area as a measure of left atrial dilation and found LAV to have a stronger association with PDA and was greater in infants with diastolic flow reversal, a finding indicative of impaired systemic perfusion. This parameter may therefore have more accuracy in predicting hemodynamic significance. Moronta et al. [4] reported that infants with echocardiographic evidence of lower preload were more likely to respond to acetaminophen (*p* < 0.001). This suggests that higher preload, as evidenced by elevations in left ventricular output, right ventricular output, and end-diastolic volume indexed to weight, may indicate greater hemodynamic impact. No difference in ductal characteristics were noted between treatment responders and non-responders, including PDA diameter indexed to weight, PDA shape, or shunt direction, further supporting the notion that PDA evaluation requires a detailed cardiopulmonary assessment.

As the cardiovascular system undergoes significant transformations in the early postnatal period, isolated echocardiographic markers should not be the sole criteria guiding decision-making. Though certain measurements have a greater correlation with PDA severity and may be helpful in predicting outcomes, an approach that considers the entire clinical landscape should be taken.

### Treatment Options

#### Timing of Treatment

Historically, a prophylactic treatment approach was employed since it decreased risk of PDA, IVH [5], pulmonary hemorrhage [6] and surgical ligation. However, no long-term benefits, including improved neurodevelopmental outcomes, have been noted [7]. This blanket approach may unnecessarily expose infants to a medication with worrisome off-target effects without clear benefit. Additionally, early closure may be detrimental in certain populations such as those with persistent pulmonary hypertension of the newborn [8].

To limit the number of infants exposed to acetaminophen or non-steroidal anti-inflammatories (NSAIDS) while also addressing a PDA prior to onset of associated negative sequelae, early targeted treatment has been studied, most recently by Gupta et al. [9]. In this randomized controlled trial, infants born 23–28 weeks gestation started a 3-day course of either ibuprofen or placebo within 72 h of life if found to have a large PDA. On reassessment at 3 weeks of age, the intervention group was more likely to have a closed or small PDA (55.5% vs 37%). Despite this difference, the separate and composite outcomes of death or moderate or severe BPD were similar. This study was limited by a high rate of open-label therapy in the placebo group (29.8%), and the criteria for initiating open-label therapy included clinical and echocardiographic findings classically associated with hsPDA, such as inability to wean respiratory support, pulmonary hemorrhage, persistent hypotension, and evidence of ductal steal. Infants with a hsPDA therefore received treatment regardless of the group into which they were randomized, making it difficult to strictly compare intervention versus non-intervention. Though the median time to open-label therapy was 11–12 days, a time-point after which the trial intervention was completed, serial echocardiograms were not obtained to evaluate the PDA prior to initiation of open-label therapy, impeding the ability to determine duration of ductal shunting and exact timing of duct closure. Additionally, ductal diameter was emphasized when selecting infants for randomization, which has previously been found to be an inadequate marker for predicting PDA severity, and possibly exposed infants without hsPDA to treatment. One strength of this study was that half of the included infants were born < 26 weeks gestation, a population with limited inclusion in previous trials. These outcomes seen are similar to previously published data as evidenced by a meta-analysis that included 7 randomized controlled trials spanning from 2004–2022, in which the authors concluded that expectant management of a PDA achieves similar outcomes as active, medical treatment [10], but these trials had similar limitations described above.

Studies utilizing detailed hemodynamic monitoring have shown more promise. In a randomized controlled trial by Zhang et al. [11], infants received ibuprofen either according to traditional guidelines focused on clinical symptoms plus ductal diameter to weight ratio or using information from repeated, comprehensive cardiopulmonary ultrasound evaluations. Decisions regarding initial PEEP were guided by lung ultrasound, and PDA treatment was initiated if there was evidence of evolving physiology such as increasing ductal diameter or flow velocity on echocardiograms, which were performed twice daily. In the cardiopulmonary ultrasound guided group, infants had higher initial PEEP (7 vs 5, *p* < 0.0001), received earlier courses of ibuprofen (3 vs 5 days, *p* = 0.004), and had higher rates of PDA closure (90.6% vs 67.9%, *p* = 0.04) with fewer ligations (0% vs 16.2%, *p* = 0.07). There was additionally a reduced risk of moderate to severe BPD (17.9% vs 40.5%, *p* = 0.04). Though this degree of monitoring may not be feasible for many neonatal intensive care units, the outcomes are encouraging and suggest that initiation of treatment within the first 3 postnatal days may be of value if targeted to the patient population most in need.

The ability to perform comprehensive hemodynamic evaluations to tailor the care of infants in real-time with TnECHO is a valuable tool that will further enrich our understanding of the PDA. Implementation of TnECHO has resulted in a change in clinical practice in 40% of cases [12, 13], and in infants with need for vasoactive support or inhaled nitric oxide, TnECHO has led to earlier blood pressure stabilization, more varied inotrope use, and decreased mortality [14]. While TnECHO did not alter the rates of PDA treatment (54.2% vs 54%), its use decreased multiple treatment courses (31.7% vs 47.8%, *p* = 0.047) without increasing PDA-related morbidities, including BPD, pulmonary hemorrhage, and NEC [15]. This highlights the importance of continued neonatal hemodynamic research, and 2024 guidelines have clarified the scope of this practice and identified important knowledge gaps [16]. Future work should focus on developing prospective clinical trials to assess the effect of TnECHO implementation, establishing standardized echocardiographic criteria that are feasible, reproducible, and reflective of the hemodynamic impact of a PDA, and broadening TnECHO training to expand the number of neonatal hemodynamic centers.

#### Pharmacologic Management

Three medications are classically used to treat PDA– indomethacin, ibuprofen, and acetaminophen. Though efficacy is comparable, the safety profile of acetaminophen is potentially more favorable [17] and has been gaining prominence even for infants in the lowest gestational ages. Moronta et al. [4] used acetaminophen as first line in a patient population that included 39% of infants born ≤ 23 weeks gestation, and even with inclusion of one of the largest proportions of extremely preterm infants, the acetaminophen response rate was > 50% across gestational ages, except for infants at 21–22 weeks gestation, whose response rate was 47%, comparable to previously published NSAID response rates. While encouraging in terms of safety and efficacy of acetaminophen, especially for extremely preterm infants, 37% of infants in the responder group reopened their shunt, suggesting that factors outside of the prostaglandin pathway impact permanent DA closure. Additionally, despite using the TnECHO screening protocol, the authors were unable to identify any parameters that would predict response to acetaminophen, again highlighting a gap in our ability to anticipate which patients will benefit from pharmacologic treatment.

While acetaminophen appears to be an effective PDA therapy with reduced incidence of gastrointestinal bleeding and NEC compared to indomethacin and ibuprofen, questions remain regarding the risk of hepatotoxicity and the utility of serum drug monitoring. Guiletti et al. [18] found no link between acetaminophen level and likelihood of PDA closure or acetaminophen toxicity, prompting a recommendation against routine therapeutic monitoring. These results contradict previous studies that suggest a critical level of acetaminophen is necessary to ensure ductal response with one study noting no ductal closure when acetaminophen levels were < 20 mg/L [19]. However, these prior studies have been limited by inconsistencies in route of administration, timing of obtaining acetaminophen concentrations, or lack of drug monitoring altogether [19–21].

The impact of acetaminophen on fetal brain development is unknown. A prospective study by Woodbury et al. [22] documented reported acetaminophen use during pregnancy and found acetaminophen exposure, especially in the second trimester, was associated with higher scores in Attention, ADHD Problems, and Externalizing Behaviors at up to 5 years of age. However, a study from Sweden [23] that included over 2 million children and controlled for siblings, found no evidence that acetaminophen use during pregnancy was associated with certain neurodevelopmental outcomes including autism (HR 0.98, 95% CI, 0.93–1.04), ADHD (HR 0.98, 95% CI, 0.94–1.02), or intellectual disability (HR 1.01, 95% CI, 0.92–1.10). With increasing use of acetaminophen in extremely preterm infants, long-term follow-up studies should be performed to further our understanding of the impact of acetaminophen use in the neonatal period on neurodevelopmental outcomes.

Though many PDAs close with treatment, 20–30% of DAs will remain patent regardless of the medication used. This remains true despite variations in dosing regimens (100% closure rate in low-dose vs conventional-dose acetaminophen with 16.66% vs 14.28% reopening rate) [24], timing in medication administration, and repeated treatment courses [25]. Specifically, failure rates after the first, second, and third course of treatment with either ibuprofen or acetaminophen were 38%, 76%, and 92%, respectively. Repeated treatment courses may instead expose infants to unnecessary medications and prolong the exposure to a ductal shunt. One strategy, however, has shown promise. Combination therapy with both ibuprofen and acetaminophen led to a higher success rate in ductal closure and a reduced need for surgical ligation compared to monotherapy [26]. For patients who still had a hsPDA after one course of monotherapy, ibuprofen was administered at a starting dose of 10 mg/kg/dose followed by 5 mg/kg/dose orally daily for three days and acetaminophen at a dose of 15 mg/kg/dose intravenously or orally every six hours for three days. While the study was limited by inclusion of full-term infants, the PDA closed without the need for surgical intervention in 88% of patients who received combination therapy. The potential synergistic benefits of a combined protocol may be a useful strategy in centers without a surgical department or infants with contraindications to surgical or transcatheter closure.

Due to these limitations, the need for novel therapies to promote ductal closure is paramount. To elucidate why certain DAs are resistant to the prostaglandin inhibitors, Sampath et al. [27] sought to evaluate if genetic variations in the 15-hydroxyprostaglandin dehydrogenase (HPGD) gene, the enzyme responsible for prostaglandin breakdown, played a role. In this study, the presence of the minor allele, rs8752, was associated with higher rates of spontaneous ductal closure and closure after medical treatment (90% vs 70% closure rate with the other haplotypes studied). Further, using machine learning algorithms [28], another study found two single nucleotide polymorphisms (SNPs) in enzymes of the cytochrome P450 system, CYP2D6*10 and CYP1A2*1C, to be predictors of successful ductal closure. This suggests that variations in the genetic makeup of infants may predict the susceptibility of the DA to prostaglandin inhibitors. If the status of these variants could be determined prior to treatment, decisions on whether to attempt medical treatment versus pursue early procedural closure could be targeted.

While current PDA drugs target the prostaglandin pathway, excessive prostaglandin levels may not be driving all cases of PDA. Previous studies have identified numerous potential PDA drug targets outside of the prostaglandin pathway, including K_ATP_ ion channels [29]. Using a high throughput screen, Li et al. [30] identified a novel vascular-specific K_ATP_ channel inhibitor, VU0542270, capable of inducing DA constriction in isolated mouse vessels. These results are a promising step towards developing DA-selective drugs that expand the therapeutic tools available to manage PDA.

#### Transcatheter Closure

Procedural closure of a PDA is used when the PDA remains hemodynamically significant despite optimal medical management or contraindication to medical management. Two options exist– surgical ligation and transcatheter device closure. The risk of complications from surgical ligation, including pneumothorax, chylothorax, arrhythmias, and vocal cord paralysis, led to the development of the transcatheter approach. The Amplatzer Piccolo Occluder was approved by the FDA in 2019 for infants weighing ≥ 700 g with resultant wide adoption. In the Vermont Oxford Network, transcatheter closure of PDA rose from 29 to 77% from 2018 to 2022. Patients who underwent transcatheter closure compared to surgical closure had a marginally shorter length of hospital stay (0.95 (0.90–0.99)) but no difference in survival or post-hospital medical management (home oxygen or gastrostomy feedings) [31]. A single center retrospective study also found no difference in length of stay, ROP, BPD or neurodevelopmental outcomes between the two approaches [32].

Adverse events associated with transcatheter closure include residual shunt, post-ligation cardiac syndrome, hemolysis, thrombocytopenia, aortic and pulmonic stenosis and device migration. A study from France [33] evaluating post-ligation cardiac syndrome found a lower incidence in patients who underwent catheter closure compared to surgical closure (0 vs 15%, *p* = 0.012) as well as lower mortality (2 vs 17%, *p* = 0.03) and rate of major complications (2 vs 23%, *p* = 0.002). The average age at procedure was 29 days and average body weight was > 1,200 g. These results may not be reproducible in a younger or smaller population. Peng et al. [34] evaluated the short and long-term cardiac complications in a cohort of over 500 patients who underwent transcatheter closure to identify factors associated with complication risk. Aortic and pulmonary end diameter were associated with adverse events within 24 h of procedure, as was younger age at time of procedure. Transcatheter device closure has been reported to be twice as expensive as a surgical ligation, requiring use of a catheterization laboratory and the device itself, which is about $6,000. Future studies should focus on whether cost savings from transcatheter device closure manifest as decreased complications, post-discharge medical management (gastrostomy, medical oxygen) and hospital length of stay.

Efforts are being taken to minimize some of the major risks seen with catheterizations. To limit radiation exposure, anesthesia exposure, and the risks associated with transport to the laboratory, some centers are placing transcatheter devices at the bedside under echocardiographic guidance [35, 36]. New devices, such as the KA micro plug and the Medtronic microvascular plug, are also under investigation given their benefits of short lengths, softer delivery devices, and lower cost, and have shown comparable short-term outcomes as the Piccolo device [37, 38]. However, the Piccolo device is currently the only FDA approved device for PDA closure in premature infants.

Ideal timing for transcatheter closure is under investigation in the ongoing PIVOTAL trial (Percutaneous Intervention Versus Observational Trial of Arterial Ductus in Low Weight Infants), a multicenter, randomized controlled trial that will compare outcomes of extremely preterm infants with a hsPDA diagnosed by PDA score ≥ 6. Infants will either undergo percutaneous PDA closure with the Piccolo device or receive conservative management. Studies such as this are essential to further our understanding of how best to approach this procedure.

#### Conservative Management

The lack of improvement in long-term outcomes seen with PDA treatment raises the concern that the negative sequelae classically associated with PDA may instead be a consequence of prematurity, and medical or surgical intervention may cause more harm than benefit. This has led to a trend towards expectant management [39–41]. A retrospective study [42] using data from the National Institute of Child Health and Human Development Neonatal Research Network showed a decrease in PDA treatment in infants across gestational ages from 21 to 16% (*p* < 0.01) from 2012–2021. In the same period, the incidence of grade 2–3 BPD rose from 13 to 26% (*p* < 0.01) among those born at 26–28 weeks gestation. Despite this concerning finding, this association should be interpreted with caution as BPD rates increased across multiple hospitals during the study period despite differing PDA treatment strategies. Additionally, other practice changes, including decreased invasive ventilation and surfactant administration, may have contributed to the increasing rates of BPD. The dynamic relationship between PDA and BPD and the impact of prolonging shunt exposure on the premature lung warrants further evaluation, especially in light of the trend towards conservative management.

Despite the uncertainties regarding the optimal approach to PDA management that improves neonatal outcomes, supportive management strategies are an important adjuvant to decrease clinical effects of a PDA. Various ventilator strategies have been used to decrease ductal shunting– both higher PEEP and permissive hypercapnia can increase the pulmonary vascular resistance and limit the volume of transductal flow [43]. Additionally, mean arterial blood pressures have been found to significantly increase in the 8 h prior to ductal closure [OR 2.094 (95% CI 1.209–3.630) *p* = 0.008], suggesting that early hemodynamic stabilization is an important aspect of timely closure of the DA [44]. As hemodynamic status in the early postnatal period is closely related to volume status, careful attention should be placed on the initial fluid and electrolyte management in these infants. Ensuring hemodynamic stability in a patient population prone to hypotension and adrenal insufficiency is critical as is providing adequate nutrition and fluid intake for growth. This is a delicate balance as lower total fluid intake in the first day of life has been associated with an increased rate of spontaneous ductal closure (90 vs 110 ml/kg/d, *p* = 0.033) [45]. Further, in the 2–3 days preceding a diagnosis of hsPDA, infants who on average received less total fluid were more likely to experience spontaneous ductal closure (118 vs 133 ml/kg/d, *p* < 0.0001). This clinical correlation is supported by Moronta et. al [4] who found that in infants < 27 weeks gestation whose echocardiogram was characterized by markers of lower preload, suggestive of lower overall total fluid, the response rate to PDA treatment was higher (*p* < 0.001).

A notable contributor to fluid status is sodium administration. Hypernatremia is common and is due to a high degree of insensible free water losses, an immature kidney that is less efficient at excreting sodium, and iatrogenic sodium administration. This can result in clinical implications with regards to a PDA. In a population of infants born < 32 weeks gestation [46], in infants with higher cumulative sodium intake in the first week of life, the risk for surgical ligation was increased [OR 1.15 (95% CI 1.10–1.21), *p* < 0.001]. Interestingly, in a subset of these patients from 23–26 weeks gestation, around 50% of their fluid intake in the first few days of life was from both volume expanders and flushes for medications and central lines, all of which were saline solutions. While easy to overlook, these sources of sodium contribute significantly to overall sodium load and can be modified by utilizing alternate solutions. Additionally, when volume resuscitation is necessary in the first day of life, other methods of volume expansion, such as packed red blood cell transfusions, should be considered as a higher hematocrit on day of life 1 has been associated with a higher likelihood of spontaneous ductal closure (44.56 vs 39.37, *p* = 0.03) [45]. These studies highlight the importance of providing thoughtful care to each infant as common practices in the NICU carry the risk of unintended consequences.

Further studies are critical to deepen our understanding of off-target effects of commonly administered medications in the NICU. In a systematic review and meta-analysis by Trindade et al. [47], caffeine administration at < 24 h of age was shown to significantly decrease the risk of PDA (OR 0.71, 95% CI 0.55–0.92) when compared to first administration at 48 or 72 h. On the contrary, aminoglycosides are associated with increased risk for PDA in both mouse models and clinically [48]. As other antibiotics have similar efficacy in the empiric treatment for sepsis [49], consideration of the side effect profile and effects on the DA could help guide clinical decision making in infants at greatest risk for PDA. Additionally, the H2 receptor antagonist, cimetidine [50], and the loop diuretic, furosemide, have been associated with PDA. Though recent data has suggested that furosemide exposure has no impact on the rate of spontaneous ductal closure (92% vs 100%, *p* = 0.384) or the post-menstrual age at which ductal closure occurs (35.0 vs 34.1 weeks, *p* = 0.558), this study only included surviving infants, and of those who expired prior to the end of the study, nearly half of the causes of mortality could be attributed to PDA [51]. Interestingly, other medications in these same classes, including famotidine and bumetanide, have not shown the same vasodilatory effect on the DA in mouse models [50, 52] and could therefore be better therapeutic options in the right clinical scenario.

A cautious approach to medication use during pregnancy is also vital. Prenatal exposure to H2 receptor antagonists or proton pump inhibitors poses a greater risk for the infant to develop a PDA [53]. Additionally, infants who were IUGR (16.9% vs 2.9%, *p* = 0.043) or born to mothers with hypertension (30.3% vs 5.9%, *p* = 0.014) were more likely to respond to PDA treatment [4], which suggests that chronic hypoxic conditions for the fetus could facilitate ductal closure postnatally. While these factors cannot be readily altered, understanding each infant’s history, underlying risk for PDA, and likelihood of treatment response could prove useful. It is crucial to gain a deeper understanding of how common medications administered during pregnancy and the neonatal period can alter the sensitive balance of factors affecting ductal tone. Continued studies focused on the relationships between medications and PDA are necessary.

## Conclusions

The pendulum for approaching PDA has shifted from high rates of prophylaxis towards minimization of medical and surgical intervention, but a balance between these two strategies is achievable. Timely treatment of PDA in a targeted patient population who would most benefit from removal of their ductal shunt should be a focus of neonatal care. Though questions remain regarding best practice with current data limited by differing stratifications of PDA severity, high rates of open-label treatment, and insufficient numbers of infants at 22–24 weeks gestation, advancements in TnECHO promise a targeted approach and adoption of a shared definition of hemodynamic significance. Along with continued research into the effects that prenatal and postnatal medication exposures have on ductal smooth muscle cells and endothelium, the path towards individualizing care for PDA management is clearer. However, the lack of variety of medications to address a PDA is noteworthy, and new approaches to PDA treatment, both at the genetic and molecular level, are vital. To fill the current knowledge gaps in PDA management, attention should be placed on the inclusion of infants < 24 weeks gestation, improvements in percutaneous transcatheter occlusion devices with trials focused on optimal timing of this intervention, continued appreciation and understanding of the off-target effects of commonly prescribed medications, and emphasis on the development of novel drugs.

## Key References

- Chesi E, Rossi K, Ancora G, et al. Patent ductus arteriosus (also non-hemodynamically significant) correlates with poor outcomes in very low birth weight infants. A multicenter cohort study. *PLoS One*. 2024;19(7):e0306769. 10.1371/journal.pone.0306769
  - Findings from this study suggest that more commonly used measures to assess hemodynamic significance may be inadequate in the early postnatal period.
- Moronta SC, Bischoff AR, Ryckman KK, Dagle JM, Giesinger RE, McNamara PJ. Clinical and echocardiography predictors of response to first-line acetaminophen treatment in preterm infants with hemodynamically significant patent ductus arteriosus. *J Perinatol*. Mar 2024;44(3):379–387. 10.1038/s41372-024-01883-w
  - This study demonstrates safety and efficacy of acetaminophen in infants < 24 weeks gestation, and findings from this study suggest an infant’s likelihood of response to acetaminophen treatment may be influenced by echocardiographic markers and prenatal factors.
- McNamara PJ, Jain A, El-Khuffash A, et al. Guidelines and Recommendations for Targeted Neonatal Echocardiography and Cardiac Point-of-Care Ultrasound in the Neonatal Intensive Care Unit: An Update from the American Society of Echocardiography. *J Am Soc Echocardiogr*. Feb 2024;37(2):171–215.10.1016/j.echo.2023.11.016
  - These guidelines provide a framework for the use of targeted neonatal echocardiography in the neonatal intensive care unit and discusses the benefits, limitations, and areas where further research is still needed.

## Data Availability

No datasets were generated or analysed during the current study.
